# Treatment Disparities Among the Black Population and Their Influence on the Equitable Management of Chronic Pain

**DOI:** 10.1089/heq.2020.0062

**Published:** 2021-09-14

**Authors:** Randall W. Knoebel, Janet V. Starck, Pringl Miller

**Affiliations:** ^1^ Department of Pharmacy, University of Chicago Medicine, Chicago, Illinois, USA.; ^2^ Department of Palliative Care, University of Illinois Hospital, Chicago, Illinois, USA.; ^3^ Physician Just Equity, Bodega Bay, California, USA.

**Keywords:** racial minority, health disparities, chronic pain, pain management

## Abstract

**Introduction:** Growing evidence suggests disparities in the prevalence, management, progression, and outcomes of chronic, nonmalignant pain-related conditions, especially for African American patients.

**Objective:** The purpose of this review is to explore studied causative factors that influence the management of chronic pain among African Americans, including factors that result in disparate care that may contribute to unfavorable outcomes.

**Methods:** This narrative review is based on available literature published on this topic published within the last 10 years.

**Results:** Assessment of chronic pain is multifaceted, often complicated by patient medical comorbidities and a complex set of biopsychosocial/spiritual/financial and legal determinants. These complexities are further exacerbated by a patient's race, by provider bias, and by structural barriers—all intersecting and culminating in disparate outcomes.

**Conclusions:** A comprehensive analysis is needed to identify quality improvement interventions and to mitigate major barriers contributing to disparities in the management of chronic pain in the African American population.

## Introduction

Chronic, nonmalignant pain (CNMP) is defined as pain not associated with cancer or cancer-associated therapy that persists beyond the expected period of healing, typically three to six months. CNMP represents a public health challenge affecting more than 116 million residents in the United States per year and has an estimated $600 billion annual impact on the U.S. economy.^1–3^

There is growing evidence to suggest disparities in the prevalence, management, progression, and outcomes of CNMP-related conditions, especially for African American patients.^4^ The Patient Protection and Affordable Care Act is in an ideal position to address these disparities in health care.^5^ Almost 10 years ago, the Institute of Medicine (IOM) report, “Unequal Treatment,” laid out a broad theoretical framework to address the root causes of health care disparities within three categories: the patient, the health process, and the health system.^6^ The authors contend that many of these discrepancies result from the interplay of a range of overlapping factors—found in Green's theoretical framework—such as barriers to trust in the doctor/patient relationship (related to communication), crosscultural awareness, compassion, empathy, sensitivity, and competence.

In an ideal situation, treatment decisions are informed by evidence, unbiased by characteristics such as sex, race, socioeconomic status, education level, and other factors (except where relevant differences are established and ameliorated by negotiations with individual patients).^6^ It is important to expose the underlying mechanisms that prevent eliminating the disparities in the chronic pain experience and in the overall health and wellbeing of the African American population. This article highlights the biological, psychological, and social factors that contribute to racial and ethnic disparities in chronic pain care and outlines a plan to reverse the current trend and provide all patients with high-quality care for chronic pain.

## Methods

This literature review focuses predominantly on recent significant articles evaluating the main drivers of disparities in chronic pain management in African Americans. The review was conducted using the following search terms: “pain” and “ethnic or ethnicity or cultural or race or sociocultural or disparity or social determinants” in PubMed and Google. Only articles published in the past 10 years were included in the review. Recent literature was accessed through The University of Chicago's Crerar library; abstracts were evaluated to determine relevance for inclusion in the review. Also included is literature focused on the patient, the provider, structural influences on communication, and access to multimodal chronic pain care. A summary of key articles is included in Table 1.

**Table 1.** Summary of key articles

Citation	Topic addressed	Key findings	No. of subjects	Demographics	Site/context
Campbell et al.^9^	Influence of race and ethnicity on pain physiology	Lower levels of pain tolerance among AA subjects resulting in more intense pain and unpleasantness compared with NHW subjects	*N*=120 *n*=62 AA; *n*=58 NHW	Healthy young adults	Study examined ethnic differences in responses to multiple experimental pain stimuli
Hoffman et al.^12^	Impact of implicit/explicit bias on pain assessment	Laypeople who strongly endorsed false beliefs reported lower pain ratings for black subjects versus white subjects. Medical students/residents that endorsed false beliefs recorded lower pain ratings and ultimately suboptimal treatment recommendations for black patients versus white patients	*n*=92 Layperson *n*=222 Medical students/residents	White, born in the United States, native English speaker	Surveyed white laypeople and medical students posing various false stereotypes to determine their baseline levels of bias and impact on pain assessment
Mathur et al.^13^	Impact of implicit/explicit bias on treatment	The study found that implicitly primed participants tended to perceive and respond more to European American patients than to AA patients	120 AA 204 European American	Medical students	Study participants were read 10 case reports describing pain severity and symptoms. Racial priming was then used to identify the ways in which automatic (implicit) and deliberate (explicit) racial biases might influence their treatment
Hirsh et al.^14^	Impact of implicit/explicit bias and contextual ambiguity on pain management	The findings suggest that clinical ambiguity—that is, discordance between patient complaints and physical exams—influenced providers' decisions to treat pain for NHW patients but not for black patients	*N*=129	Medical residents and fellows	Subjects were asked to make clinical decisions on 12 unique patient-simulated cases, evaluating each patient's pain level and the likelihood of using different analgesics
Beach et al.^15^	Patient–provider communication	Providers were more dominant in conversations with black patients compared with white patients. Black patients were significantly less talkative than white patients during their examinations and provided less information in both the psychosocial and biomedical domains	*N*=354 patient–provider encounters	Black and white HIV-infected patients	Patient–provider encounters coded with the Roter Interaction Analysis System across four HIV care sites in the United States
Anderson et al.^16^	Patient–provider communication	Thirty-one percent of the AA patients received analgesics of insufficient strength to manage their pain. Seventy-four percent of physicians underestimated pain severity for AA patients	*n*=108 *n*=55	AA and Hispanic cancer patients Physicians and nurses who treat these patients	Completed a survey about their pain intensity, pain interference, and attitudes toward analgesic medications. Completed a questionnaire regarding cancer pain and its management in their practice settings
Hsieh et al.^17^	Impact of race concordance on pain assessment	When patient–provider race were concordant patients were more likely to exhibit more distressing pain behaviors	*N*=102	Race concordant (*n*=52), or Race discordant (*n*=50)	Participants were exposed to a cold pressor task under 1 of 2 conditions: Race-concordant OR non-race concordant
Bach et al.^20^	Structural barriers to effective pain care	Twenty-two percent of physicians provide care for 80% of AA in the United States and these physicians report limited access to health care resources, such as specialists and diagnostic imaging	*N*=150,391 patients; 4355 primary care physicians	Medicare beneficiaries for medical “evaluation and management”	Cross-sectional analysis evaluating patients' visits who were seen by primary care physicians who participated in a biannual telephone survey
Varkey^21^	Structural barriers to effective pain care	Clinics serving at least 30% minority patients have less access to medical supplies, fewer examination rooms per physician, fewer referrals to specialists. more likely to be covered by Medicaid, and more medically and psychologically complex. Physicians at these clinics report less control over their work environments, lower job satisfaction levels, and higher rates of burnout	Ninety-six clinic managers, 388 primary care physicians, and 1701 of their adult patients	Hypertension, diabetes mellitus, or congestive heart failure	Cross-sectional study comparing clinics with >30% underrepresented racial minority versus those with <30%
Gebauer et al.^23^	Structural barriers to effective pain care	Sixty-three percent of residents in low-nSES areas were more likely to receive opioid-only therapy and not receive referrals for physical therapy. In contrast, patients in high-nSES areas tend to receive both opioid and physical therapy	*N*=1646	54.7% white; 67.9% female Average age 55.7 years	Influence of n-SES on management of low-back pain evaluating NSAIDS, opioids, physical therapy referral/initiation
Joynt et al.^24^	Structural barriers to effective pain care	Opioids were prescribed more frequently at visits from patients of the highest SES quartile compared with patients in the lowest quartile. Black patients were prescribed opioids less frequently than white patients across all measures of SES.	*N*=50,236 patient visits	12%> 65 years of age; 24% Black race; 22% from neighborhood with >20% poverty	Data from the National Hospital Ambulatory Medical Care Survey evaluating the prescribing of opioids to patients presenting with moderate-to-severe pain
Scholl et al.^31^	Opioid overdose deaths and race	Opioid-related mortality is affecting whites and blacks equally. Blacks experiencing the largest relative increase (25.2%) in opioid-involved deaths from heroin and synthetic opioids (often laced with heroin)	In 2017, among 70,237 drug overdose deaths, 47,600 (67.8%) involved opioids	Increases across age groups, racial/ethnic groups, county urbanization levels, and in multiple states	Data from United States National Vital Statistics System, Mortality file

AA, African American(s); NHW, non-Hispanic white(s); NSAIDS, non-steroidal anti-inflammatory drugs; nSES, neighborhood socioeconomic status.

## Literature Review

### Influence of race and ethnicity on pain physiology

Pain is a biopsychosocial phenomenon. Simply put, one's pain perception, affective response to pain, and ultimate pain behavior is influenced by a complex set of biological, psychosocial, and cultural interactions.^7^ Pain, therefore, is largely subjective, highly individualized, and often defined by “what the patient says it is.” Physiologically, pain results from a nociceptive afferent impulse relayed from the periphery to the somatosensory cortex, which triggers the sensation of pain. This same impulse relays to limbic structures resulting in an emotional response.^8^ Racial differences in pain perception and responses are documented in experimental models. The results indicate significantly lower levels of pain tolerance or thresholds among African American subjects resulting in more intense pain and unpleasantness compared with non-Hispanic white subjects.^9^ This suggests that pain disparity may be related to racial and ethnic differences in pain processing and modulation caused by genetic polymorphisms. These biological differences likely contribute to the difficulties in managing pain across racial and ethnic groups.

Environmental, social triggers, and resultant allostatic load also have an impact on pain. Specifically, they influence gene transcription through gene methylation and epigenetics, suggesting that health outcomes may be less dictated by genes and more by environmental and social triggers.^3^ Consider, for example, Mukherjee's formula: phenotype (what we are)=genotype (gene)+environment+triggers+chance.^10^ Also, emerging evidence on the nature versus nurture debate reveals that psychosocial factors such as mood, rumination, and catastrophizing can significantly influence the pain experience.^11^ Therefore, a thorough assessment of CNMP requires consideration of the impact of nonbiological factors on racial and ethnic groups.

### Influence of race in doctor/patient communications

Doctor/patient communications and trust may also be a significant factor in pain analyses.^3^ For example, determining how bias—either implicit or explicit—influences one's perception of pain and response to pain is important in analyzing pain levels. Hoffman et al., in two independent studies, set out to examine the influence of racial biases on responses to pain, specifically as they relate to false beliefs about biological differences between blacks and whites and vice versa—essentially, biology influencing responses to pain.^12^ In the first study, the authors surveyed white laypeople, posing various false stereotypes to determine their baseline levels of bias. Survey participants who strongly endorsed false beliefs about biological differences reported lower pain ratings for black subjects versus white subjects.^12^ In the second study, the authors surveyed white medical students and residents, half of whom expressed the same false beliefs. The result was lower pain ratings and ultimately suboptimal treatment recommendations for black patients versus white patients.^12^ Study participants who did not endorse these beliefs rated black patients' pain levels higher than white patients’, and showed no bias in treatment recommendations.^12^ The authors' conclusion: Lay people and individuals with limited medical training might hold false beliefs about biological differences between black and white patients. When such false beliefs inform management decisions it may contribute to racial disparities in pain evaluation and management.^12^

In another study, Mathur et al. evaluated 120 self-identified African American medical students and 204 European American medical students. Study participants were read 10 case reports describing pain severity and symptoms.^13^ Racial priming was then used to identify the ways in which automatic (implicit) and deliberate (explicit) racial biases might influence their treatment.^13^ Before reading the case reports, half of the students were implicitly primed through a 100-millisecond flash image of an African American or European American; the remaining students were explicitly primed through a 7-sec flash of the same image.^13^ The study found that implicitly primed participants tended to perceive and respond more to European American patients than to African American patients when the effect of patient race was presumably below the level of conscious control or regulation.^13^ The opposite effect was observed when the patient's race was presented explicitly, such that participants perceived and responded more to the pain of African American patients than to European American patients.^13^ This finding is likely the result of participants' conscious efforts to respond without prejudice or bias.

These results suggest that stereotypes, rather than general racial biases, may be responsible for the observed race-based differences in pain perception and response. The study findings present two new questions: How pervasive are these implicit racial biases or stereotypes? And what else might be at play in assessing pain disparities? To answer these questions, Hirsh et al.^14^ set out to examine the role of provider bias and contextual ambiguity in the care of white and black patients suffering from acute pain. Medical residents and fellows recruited from across the country were asked to make clinical decisions on 12 unique patient-simulated cases, evaluating each patient's pain level and the likelihood of using different analgesics. The findings suggest that clinical ambiguity—that is, discordance between patient complaints and physical exams—influenced providers' decisions to treat pain for white patients but not for black patients.^14^ The study authors provided two potential interpretations of this finding: (1) black patients received care that was less responsive to contextual information, and (2) black patients received more consistent care while white patients received variable care and in some instances were overtreated.^14^

The Hirsh study highlights the complexity of patient, provider, and contextual factors on pain management decisions, and specifically on provider/patient communications. Furthermore, while implicit bias seems to influence first impressions, if the case presented is clear and unambiguous then these initial impressions seem to play a lesser role. Therefore, the quality of patient/provider interactions appears to be an important factor in assessing and addressing disparities in chronic pain management.

Furthermore, a study comparing patient/provider communications among black and white HIV-infected patients found that providers were more dominant in conversations with black patients compared with white patients.^15^ The differences were largely determined by observation—that black patients were significantly less talkative than white patients during their examinations and provided less information in both the psychosocial and biomedical domains.^15^ This concept of ambiguity and engagement of black patients during examinations is confirmed in a study by Anderson et al.^16^ Anderson et al. found that inadequate pain assessment and staff members' limited knowledge of pain management in cancer patients is a significant barrier to physicians' and nurses' ability to manage cancer pain in low-income minority patients.^16^

How do provider/patient interactions differ when provider and patient are the same race? To answer this question, Hsieh et al. evaluated patients' reporting of pain using severity ratings, measurement of affects, and nonverbal pain behaviors. Hsieh et al. compared the differences when provider and patient race were concordant and when they were not.^17^ They found that while patients may rank their pain severity the same, in situations with race concordance, patients are more likely to exhibit more distressing pain behaviors.^17^ These findings suggest that interpersonal and sociocultural behaviors between patient and provider are crucial to determining true pain levels, with implicit bias likely to occur on both sides of the relationship.

Additionally, health illiteracy may contribute to the overall effectiveness of provider/patient interactions, especially when considering that only 12% of Americans have proficient levels of health literacy.^18^ Identifying techniques to encourage provider/patient interactions may help reduce disparities. Engaging patients in their own care, particularly those with chronic diseases, is essential to reaching optimal outcomes.^19^ What is clear is that the assessment of chronic pain is complicated, primarily because pain is inherently subjective, and a lack of objective data or diagnostic concordance complicates comprehensive assessments. The primary means to assess pain remains dependent on patient self-reports and providers with the clinical acumen and communication skills to establish rapport and build trust with their patients. Not surprisingly, provider/patient interactions are highly susceptible to provider bias and variations in quality.

### Influence of race on structural barriers to effective pain care

In addition to inadequate provider/patient communications, other health system-related factors such as access to care may be driving disparities in pain management. Bach et al. investigated the role that access to care plays in driving disparities.^20^ They found that just 22% of physicians provide care for 80% of African Americans in the United States and that these physicians report limited access to health care resources, such as specialists and diagnostic imaging.^20^ Bach et al. found that physicians caring for black patients are less likely to be board certified and more likely to report an inability to provide high-quality care to all of their patients.^20^ Varkey and colleagues took the Bach et al.^20^ study one step further, evaluating the workplace characteristics of primary care clinics, in which at least 30% of patients are underrepresented racial or ethnic minorities.^21^ They found that these clinics have less access to medical supplies, fewer examination rooms per physician, and refer fewer patients to specialists.^21^ These patients are more likely to be covered by Medicaid, report symptoms of depression (within the previous 2 weeks), and convey lower levels of health literacy.^21^ Also, physicians at these clinics tend to perceive their patients as speaking little or no English, having more chronic pain and substance use disorders, and being more medically and psychologically complex.^21^ Furthermore, clinics with at least 30% underrepresented minority patients are generally more chaotic. Physicians at these clinics report less control over their work environments, lower job satisfaction levels, and higher rates of burnout.^21^ The study authors' conclusions: “The combination of time pressure, insufficient resources, and complex patients is likely to constitute a ‘perfect storm’ of challenges that physicians face in providing quality care to large proportions of minority patients.”^21^

A study by Azhar et al. evaluated access and referrals to specialty pain and palliative care services for patients with advanced cancers.^22^ The authors observed that patients with limited or no insurance had significantly higher pain scores and tended to be young, single, non-white, and often on opioids.^22^ These findings reinforce the barriers to access that hinder optimal chronic pain management, including lack of a well-trained and culturally competent workforce.

Neighborhood socioeconomic status (nSES) is another factor that can significantly influence the quality of care delivered to patients in pain. In a study by Gebauer et al., the influence of nSES was evaluated based on the type of treatment patients with new back pain receive in primary care clinics.^23^ The team observed that 63% of residents in low-nSES areas were more likely to receive opioid-only therapy and not receive referrals for physical therapy. In contrast, patients in high-nSes areas tend to receive both opioid and physical therapy.^23^ This finding is in sharp contrast to that of Joynt et al., which notes a significant decrease in opioids prescribed for moderate-to-severe pain.^24^ However, this study is based on emergency room approaches to care, which are vastly different from primary care approaches. Gebauer et al., suggest that the low nSES and increased opioid prescriptions might be attributed to patients' lower education levels and a certain unwillingness to accept nonpharmacologic or non-narcotic treatment modalities.^23^ However, limited access to comprehensive pain care—including rehabilitative medicine, integrative medicine, and pain specialty consultations—and minimal patient/provider communications also contributes to the “non-guideline, quick-fix” approach. Additionally, insufficient neighborhood resources often lead to physical and social isolation and increased stress that often proves detrimental to the chronic pain experience.^25^

Differences in pain management also appear to be age independent. For example, in the Emergency Department (ED), black children with appendicitis are less likely to receive pain medication for moderate pain or opioids for severe pain compared with white children.^26^ Another study observed that in outpatient nonemergency settings, white children are more likely to receive opioids while minorities are more likely to receive nonopioid analgesics.^27^ Recent evidence suggests three main drivers of high-risk opioid prescribing to Medicaid enrollees—white race, rural residence, and depression.^28^ Also, based on the reviewed evidence, blacks are less likely to receive an opioid for acute pain (nonguideline based), more likely to receive an opioid for chronic pain (nonguideline based), and less likely to receive a high-risk opioid prescription (unless depressed), due to concern over misuse. The bias around African Americans and opioid use disorders or addictions is not supported by the national demographics of opioid users.

### Influence of race and the opioid epidemic

Historical patterns of opioid use from 1993 to 2009 show a disparity between the rate of prescription opiates prescribed to white Americans (∼16/100,000) and the rate prescribed to African Americans (∼7/100,000). These values directly correlate with opioid overdose rates for white Americans (∼15/100,000) and African Americans (∼5/100,000).^29^ Om makes an interesting point, stating that the present national attention on the opioid epidemic might be in part because of past failures in the treatment of addictions. However, it is also plausible that the reaction to the opioid epidemic is yet another example of the disparate attention given to diseases based on the demographics they affect.^30^

Additionally, more recent statistics suggest that opioid-related mortality is affecting whites and blacks equally. This is mainly driven by heroin and synthetic opioids (often laced with heroin), with blacks experiencing the largest relative increase (25.2%) in opioid-involved deaths.^31^ Heroin rates are currently increasing at 31% (95% confidence interval [CI]: 27% to 35%) per year for whites and 34% per year for blacks (95% CI: 30% to 40%), respectively.^32^ Concurrently, synthetic opioids are increasing at 79% (95% CI: 50% to 112%) for whites and 107% (95CI: −15% to 404%) for blacks.^32^

Historically, prescription opioid drugs have been the gateway to heroin use. This might suggest that heroin use among African Americans results from shortfalls in pain evaluation and management, affordability, and easier access to heroin than to prescription opioids.^33^ Additionally, some minority patients face structural barriers and limited availability of opioids in their neighborhood pharmacies due to “medication deserts.”^34^ Altogether, these factors illustrate that disparities in access may be driving the current opioid epidemic.

## Findings

In this article, we explored the patient-, provider-, and system-level drivers of racial disparities. These disparities represent an ethical dilemma as well as practical barriers that further perpetuate racial differences. The problems are both broad and complex. As health care providers, it is our professional responsibility to equitably deliver evidence-based management that is individualized and patient concordant. Because race has both practical and societal implications, we must address all barriers that prevent us from achieving optimal chronic pain management. Furthermore, it is a mistake to focus on genetic contributions to racial disparities. Doing so minimizes the impact of environmental, structural, and societal factors on managing chronic pain. More importantly, racial bias can lead to stereotyping that detracts from employing objective clinical decision making to treat chronic pain.

Racial bias plays a role in some provider tendencies to minimize the pain suffered by African Americans (typically providers with limited medical training rather than those with more extensive medical training), assuming little ambiguity in the case.^12,14^ Focusing on provider/patient communications will require more comprehensive cultural competency training to overcome this disparity gap.^14–16,19^ A number of structural barriers exist to prevent standard multimodality pain management in minority patients—including access to specialists (i.e., board-certified anesthesia pain and palliative medicine physicians), diagnostic imaging, supplies, integrative medicine, psychosocial counseling (i.e., cognitive behavioral therapy), and the full array of pharmaceuticals. Both neighborhood and clinic-specific dynamics create time-constrained and chaotic care environments that perpetuate implicit racial disparities and result in low-quality or nonguideline pain management.

African Americans are not as directly impacted by the overprescribing of prescription opioids, because of the limitations we have addressed so far. However, recent data suggest that African Americans die from heroin and synthetic opioids at a similar rate as their Caucasian counterparts die from prescription opioids. As with other chronic conditions, chronic pain when managed poorly has adverse long-term sequelae such as: impaired sleep, cognitive processes, and brain function; mood/health; cardiovascular health; sexual function; and overall quality of life.^35^ Furthermore, chronic pain can become increasingly more complex over time resulting in treatment-refractory disease—thus making a timely referral and implementing an early multimodal management plan critical.^35^ Taken together, these data support a quality improvement initiative to focus more resources on managing chronic pain in the African American population and to confer a patient and societal benefit.^35^

## Discussion

While experimental models have demonstrated biological and physiological difference in pain perceptions between Black and white patients the etiology is not well described (9), it is also unclear whether this experimental difference has applicability to clinical practice and the allocation of therapeutic interventions for the treatment of acute and chronic pain. Therefore, recruiting more African Americans into clinical research studies will help clarify these variables. Yet, already, our recruitment efforts are compromised by the general mistrust minorities have for the medical establishment.^36^ One way to overcome this mistrust is to partner with community organizations to bring more value to these communities. Community instability or neighborhood social environment has been demonstrated to significantly influence the degree of mistrust in the health care system among the African American community.^36^ By developing shared goals and building alliances with African American communities, we have an opportunity to increase trust with our neighbors.

### Empathy can be taught

Empathy has been shown to have a positive impact on the quality of patient/provider interactions. Yet many primary care providers appear to lack empathy, especially when evaluating patients with chronic pain.^37^ This lack of empathy, or at least the appearance of such, highlights a known concern about inadequate training in pain assessment that may focus more attention on pain-related dysfunction in general.^38^ The data on pain-assessment skills emphasize a need for empathy and, potentially, deeper cultural training for all health care professionals. As patients become more diverse, cultural competence and freedom from bias become crucial professional responsibilities. A related issue is the mismatch between the diversity of health care professionals and patients, as minorities make up 25% of the U.S. population and only 10% of the health care profession.^38^ A more diverse workforce will enhance cultural sensitivity and the quality of patient interactions while also strengthening the medical research agenda. Culturally focused quality improvements will inform a more enlightened health care system.^6,39^

### The chronic care conundrum

The spectrum of health literacy affects the care being delivered and warrants a comprehensive educational campaign. In 2012, the IOM published “Ten Attributes of Health Literate Health Care Organizations,” to help people navigate, understand, and use available information and services to take control of their own health.^40^ Health care organizations that exemplify these 10 attributes are better able to help patients access and benefit from a range of health care services necessary for comprehensive CNMP.^40^ One study found that black or African American patients who received an opioid prescription in the past year not only had higher depressive symptoms but were also associated with increased health care utilization, including visiting low-income clinics for help with chronic pain.^41^ Based on these findings, the authors suggested that focusing on patients' psychosocial factors would likely confer a larger benefit on CNMP outcomes for patients receiving care at low-income clinics.^41^ Unfortunately, as published by Varkey, the challenging work environments at low-income clinics contribute to treatment and resource disparities. Burdened by time pressures to see patients and demands to increase relative value units, providers are hampered in their ability to accurately assess the presenting symptoms of under-represented minority patients, especially when there are cultural or language barriers. While these pressures exist in many clinics, low income or not, when added to other resource limitations, providers suffer from stress, fatigue, and burn out that can further trigger a lack of empathy and lead to implicit bias.

### A team-based approach to chronic pain

Clearly, the work environment has a role to play in health disparities, particularly in chronic pain management. One solution is to employ targeted interventions designed to address clinic chaos, work controls, and physician burnout. This is even more relevant today as the U.S. faces a primary care physician shortage. The solution may reside in full implementation of a team-based approach to care—delegating tasks to team members—thus addressing the primary care shortage without adding new physicians.^42^ The team composition consists of physicians, advanced practice nurses, physician assistants, and nurses.^42^

Pharmacists are also an important part of the team-based approach to chronic pain management. Most already have relationships with patients in the community, are crucial members of medication management teams, and have the required expertise to review medications and educate patients.^43^ Research demonstrates that pharmacists with advanced clinical training perform at least as well as physicians in managing chronic disease states, measured by intermediate outcomes such as glycemic controls and blood pressure.^44^ Clinical pharmacists who support the management of complex pain patients can reduce burdens on physicians and better guide concordant opioid-based pain care.^45^

## Time for a Paradigm Shift

In *The 7 Habits of Highly Effective People*, author Steven Covey recounts how people see the world, … “not as it is, but… as we are conditioned to see it.”^46^ Paradigms are the sources of our attitudes, behaviors, and relationships with others.^46^ If we want major changes, we must transform the way we look at things, and part of what defines a paradigm shift is learning new habits.^46^ “Reducing racially or culturally based inequities in medical care is a moral imperative,” explains Geiger.^47^ As health care professionals, we must lead by example. We must encourage societal change by taking the first important step of honest self-reflection—not only acknowledging the need for change but also providing the solutions. We can start with implicit bias, which is likely the most common form of bias among health care providers and contributor to observed patterns of inequities in the receipt of high-quality care.^48–50^ Implicit bias is universal, often subconscious, and even the most well-meaning clinicians harbor deep-seated biases, which affect medical decision making and the quality of communication and nonverbal behavior.^50^

There is emerging evidence that a variety of social psychological interventions may reduce implicit bias. In one study, implicit biases were viewed as deeply engrained habits that can be replaced by specific behavioral strategies, including stereotype replacement, counter-stereotype imaging, individuation, perspective taking, and increasing interracial contact.^51^ More research is needed to demonstrate that changes in implicit bias are linked with reduction in discriminatory behaviors and improvement in health equity. There is also a need to recognize the ways in which policies and procedures in medical and social institutions sustain racial inequality. While awaiting both the dismantling of institutional legacies of implicit bias and the corroborating research on individual implicit bias reduction, we propose a course of action to begin to mitigate their effects.

(1) Acknowledge the pervasive presence and pernicious effects of implicit bias.
(2) Avoid stereotypes; deploy targeted strategies such as stereotype replacement using a consciously adjusted response or counter-stereotypic imaging in which the patient is framed as the stereotypic opposite.
(3) Adopt an “individuation” approach with focus on each patient's unique personal history and context for their care.
(4) Empathize with each patient—incorporate cognitive empathy of “putting yourself in your patient's shoes” and affective empathy of sharing in the experience of their illness and pain.
(5) Establish meaningful partnerships in which the patient/provider exchange is a collaboration between equals and forms the basis of shared decision making.
(6) Engage in ongoing critique of our behaviors, attitudes, and biases through patient feedback and self-reflection.

It is time for our paradigm shift.

## Abbreviations Used

AA: African American(s)
CI: confidence interval
CNMP: chronic, nonmalignant pain
IOM: Institute of Medicine
NHW: non-Hispanic white(s)
NSAIDS: non-steroidal anti-inflammatory drugs
nSES: neighborhood socioeconomic status
